# Rapid Simultaneous Determination of Paraquat and Creatinine in Human Serum Using a Piece of Paper

**DOI:** 10.3390/mi9110586

**Published:** 2018-11-12

**Authors:** Tsui-Hsuan Chang, Kuo-Hao Tung, Po-Wen Gu, Tzung-Hai Yen, Chao-Min Cheng

**Affiliations:** 1 Institute of Biomedical Engineering, National Tsing Hua University, Hsinchu 300, Taiwan; allmyown74@gmail.com (T.-H.C.); t19872@hotmail.com (K.-H.T.); 2 Department of Laboratory Medicine, Chang Gung Memorial Hospital and Chang Gung University, Linkou 333, Taiwan; trent12368@gmail.com; 3 Department of Nephrology, Clinical Poison Center, Kidney Research Center, Center for Tissue Engineering, Chang Gung Memorial Hospital and Chang Gung University, Linkou 333, Taiwan

**Keywords:** paraquat, creatinine, paper-based analytical device

## Abstract

Paraquat intoxication is characterized by acute kidney injury and multi-organ failure, causing substantial mortality and morbidity. This study aims to develop a 2-in-1 paper-based analytical device to detect the concentrations of paraquat and creatinine in human serum, which can help clinicians diagnose patients with paraquat poisoning in a more rapid and geographically unrestricted manner. The procedure involves fabrication of a paper-based analytical device, i.e., printing of design on a filter paper, heating of wax-printed micro zone plates so as molten wax diffusing into and completely through the paper to the other side, forming hydrophobic boundaries that could act as detection zones for the paraquat colorimetric assay, and finally analysis using ImageJ software. The paper employed a colorimetric sodium dithionite assay to indicate the paraquat level in a buffer or human serum system in less than 10 min. In this study, colorimetric changes into blue color could be observed by the naked eye. By curve fitting models of sodium dithionite in normal human serum, we evaluated the serum paraquat levels for five paraquat patients. In the sodium dithionate assay, the measured serum paraquat concentrations in patients 1–5 were 22.59, 5.99, 26.52, 35.19 and 25.00 ppm, respectively. On the other hand, by curve fitting models of the creatinine assay in normal human serum, the measured serum creatinine concentrations were 16.10, 12.92, 13.82, 13.58 and 12.20 ppm, respectively. We found that the analytical performance of this device can compete with the standard of Clinical Laboratory of Chang Gung Memorial Hospital, with a less complicated sample preparation process and more rapid results. In conclusion, this 2-in-1 paper-based analytical device has the advantage of being simple and cheap, enabling rapid detection of paraquat intoxication as well as assessment of renal prognosis.

## 1. Introduction

Paraquat (N, N′-dimethyl-4, 4′-bipyridinium dichloride; PQ) is a highly effective, low-cost, and easily accessible herbicide [1]. It is used widely throughout the world and is especially prevalent in developing countries. Its ubiquity and pervasiveness in developing countries is especially alarming, with little to no regulations despite its lethality even in small doses [2,3]. The easy availability and high toxicity of paraquat explain its common use to commit suicide. Reports by the World Health Organization noting the global prevalence of acute pesticide poisoning as a means of suicide have resulted in significantly greater attention and efforts to remedy this situation [4,5]. Among pesticides, paraquat is a highly toxic agent, which has a mortality rate of 60%–80% [6,7,8]. The mechanisms of PQ intoxication begin with the generation of superoxide anions. Subsequent reactive steps lead to the formation of toxic reactive oxygen species, and the oxidation of cellular NADPH (nicotinamide adenine dinucleotide phosphate). This is the main source of reducing equivalents for intracellular reduction in PQ poisoning, which then results in the disruption of important NADPH-dependent biochemical reactions [9]. PQ intoxication mortality is attributed to respiratory failure resulting from oxidative insult to the alveolar epithelium, and subsequent obliterating fibrosis or acute respiratory distress syndrome [10].

Serum PQ concentrations have been used to diagnose the severity of poisoning [11,12]. Patients may recover if their serum PQ concentrations remain under 0.3 ppm at 10 h after poisoning [13]. The severity index of paraquat poisoning (SIPP), an index for clinicians to determine the severity of PQ intoxication, is determined by multiplying serum paraquat concentration on admission (ppm) by the time to treatment (hour). SIPP values under 10 are an indication to clinicians that poisoned patients have a higher probability of survival [14]. Different approaches to measuring PQ serum concentration are available including high-performance liquid chromatography (HPLC)/mass spectrometry (MS), gas chromatography, and photometry coupled with a sodium dithionite assay [11,15,16,17,18,19]. Another option reported in the literature is the use of surface-enhanced Raman spectroscopy (SERS) [20,21,22,23]. The complexity, time consumption, and equipment demand of such approaches make them impractical, especially in resource-poor environments.

Paraquat poisoning can result in multiple organ failure that primarily affect the lungs, kidneys, heart, liver, and nervous system. As the main detoxification organ, the kidneys encounter very high concentrations of paraquat during the body’s process of elimination. This leads to vacuolization of the proximal tubular cells, resulting in acute small tube tubular necrosis, a common cause of acute kidney injury and a disease/organ failure state clinically detected by measuring creatinine levels [7,8]. Normally, creatinine concentration in the human body ranges from 5.0–10.0 ppm for women and 7.0–12.0 ppm for men [24,25], with the difference being attributable to variations in muscle as a percent of total body makeup. More muscle is correlated to higher creatinine levels. The level of onset for acute kidney injury has been defined as a serum creatinine levels greater than 15 ppm [26]. The incidence of acute kidney injury following paraquat exposure is approximately 50%. Average serum creatinine levels reach a peak at approximately five days post-ingestion, and usually normalize within three weeks [27]. Clinically, only patients who have developed kidney failure will receive ameliorative hemodialysis [26]. Typically, the clinical sequence of treatment approaches aims to determine the symptoms of severe organ failure at the time of diagnosis in order to deduce necessary treatment methods. Symptomatically, kidney failure follows low urine output or no urine output, as the kidneys fail or become compromised, and they lose their ability to regulate fluids and electrolytes and to remove waste products from the body. Early detection of serum creatinine levels is a critical step toward reducing organ failure numbers.

In recent years, paper-based analytical devices (PADs) have become increasingly well-developed, potential translational medicine platforms for disease diagnostics [28,29,30,31,32]. Using various fabrication methods such as wax patterning, inkjet printing, flexographic printing, photolithography, paper cutting, and shaping [33], paper has been used as a viable substrate material with distinct advantages. These include power-free fluid transport via capillary action, a high surface area-to-volume ratio that improves detection limits for colorimetric methods, and the ability to store reagents in active form within a natively fibrous network [33]. As with many disease and illness states, adequate treatment requires timely diagnosis. The 2-in-1 PADs we have developed are notably timely diagnostic tools capable of simultaneously determining serum PQ and creatinine levels. Detection time for our device is between 10 and 20 min, at least half the time required by expensive clinical equipment. While the diagnostic speed of our device is critically important to its usefulness, it is important to note that our device is also inexpensive (approximately 50% less than clinical methods) and easy to use, with only small specimen samples required. Because early diagnosis equates to earlier treatment, and increased patient survival rates, PADs such as ours represent a potentially life-saving alternative for first responders.

Paraquat’s unfortunate ease of availability as a herbicide makes it a simple and straightforward avenue for misuse, and farmers a primary victim of its deadly effects. If a patient must be sent from a rural medical institution to a distant, better-equipped medical facility, precious diagnostic and treatment time is consumed by transportation time. Our goal is the development of a 2-in-1 PAD that can act as the first line of treatment in emergency situations, even before ambulance transportation. Following adequate and rapid diagnosis “in the field”, transfer to a clinic equipped to provide necessary therapy would ensue in a timely and effective manner.

## 2. Materials and Methods

### 2.1. Reagents

Paraquat dichloride hydrate (Sigma Aldrich, St. Louis, MO, USA); sodium hydroxide (Sigma Aldrich); ascorbic acid (Sigma Aldrich); phosphate buffered saline (Sigma Aldrich); creatininase (Sigma Aldrich); sarcosine oxidase (Sigma Aldrich); peroxidase (Sigma Aldrich); creatine kinase (Sigma Aldrich); creatinine (Sigma Aldrich); ABTS (2,2’-Azino-bis(3-ethylbenzothiazoline-6-sulfonic acid)diammonium salt, Sigma Aldrich); Whatman qualitative filter paper No. 1 (GE Healthcare Life Science; No. 1001-150, Budapest, Hungary).

### 2.2. Fabrication of 2-in-1 PADs

**Figure 1:**
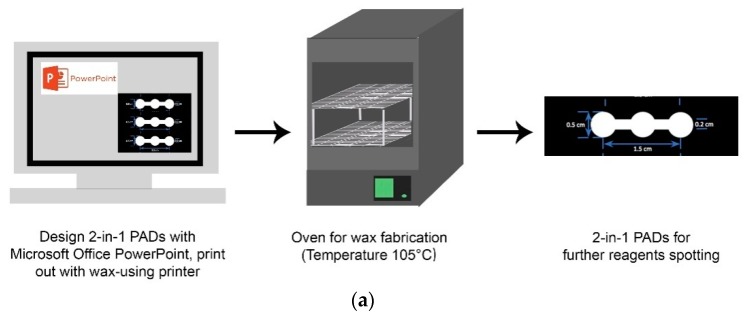

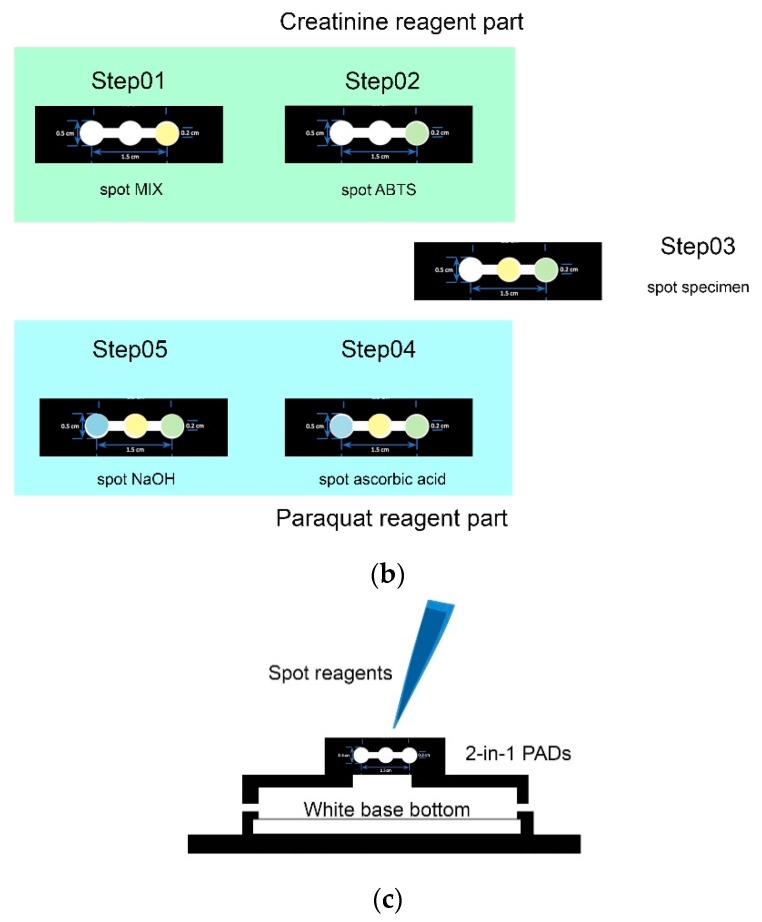
Fabrication steps of 2-in-1 paper-based analytical devices (PADs): (**a**) wax fabrication of 2-in-1 PAD design; (**b**) procedures for spotting reagents onto 2-in-1 PADs; and (**c**) device display.

We used Microsoft PowerPoint as a pattern design tool. We developed a 2-in-1 pattern with three circular sample areas, each with a diameter of 0.5 cm, and a rectangular flow channel that connected each of the sample/test zones, 0.15 cm-wide and 1.5 cm-long. The left circle acted as the PQ detection zone, the central circle as the specimen zone, and the right circle for creatinine detection. To fabricate 2-in-1 PADs, we used wax-based printing technology to print the design on Whatman qualitative filter paper No. 1 using a wax printer (Xerox Phaser 8650N color printer, Xerox Corporation, Norwalk, CT, USA). After printing, paper layers were heated to 105 °C for 5 min to melt the wax and allow it to penetrate to the other side, thus creating hydrophilic detection zones with hydrophobic wax boundaries, as shown in Figure 1a.

### 2.3. Colorimetric Assays

**Figure 2:**
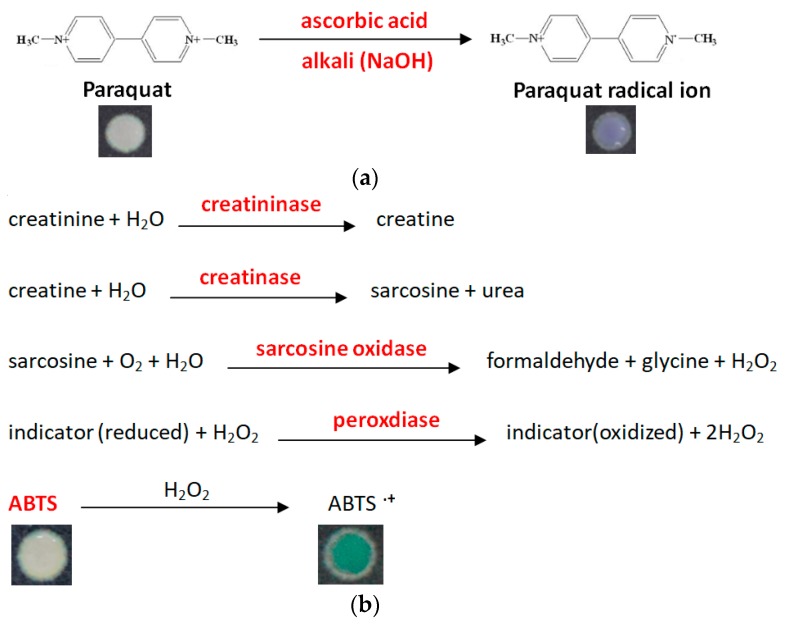
Mechanism of paraquat and creatinine assay: (**a**) paraquat mechanism in our paper-based analytical device, (**b**) creatinine mechanism in our paper-based analytical device (red text indicates the agent being added to our paper-based analytical device).

We prepared a mixture of 4 chemicals: Creatininase, sarcosine oxidase, peroxidase, and creatinine kinase (suspended in phosphate buffered saline, PBS) at a ratio of 1:1:1:1 by volume. This mixture can be preserved for over 2 weeks in a 4 °C refrigerator and still be effective. The colorimetric mechanism we used leveraged the reaction of ABTS and H_2_O_2_ rather than the Jaffe reaction because of potential creatinine component reaction interference [34]. The reagent application order is important when creating our PAD due to the timing for each reaction and the order of subsequent color changes detected by image analysis. First, we applied 4 μL of our mixture to the right circle, our creatinine zone. Our second step was the application of 4 μL of 20 mM ABTS to our creatinine zone. Our third step was the application of 8 μL of specimen to the central circle, allowing it to flow to both left and right circular zones. Our fourth step was the application of 5 μL of 10% ascorbic acid to the left circle, our PQ detection zone. The fifth and final step was the application of 5 μL of 5N NaOH to our PQ detection zone. After maintaining our device in a humid state for 10 min, we observed the PQ zone for any colorimetric change (blue) (Figure 2a). After maintaining our device in a humid state for an additional 10 min, we observed the creatinine detection zone for any colorimetric change (green) (Figure 2b). Images were preserved with a digital camera (EOS 5D Mark III, Canon, Tokyo, Japan), and then analyzed using ImageJ software (Figure 1b). Based on our experimental results, we determined that the detection zones of our device produced the best results when the device was kept in a humid environment and was not allowed to dry out once reagents and test samples were added. Furthermore, we found that keeping the device flat prevented unintended reagent and enzyme mixing, and produced the most homogeneous and low-interference images. We also found that colorimetric results were best and most uniform if the device was implemented using a white base background as depicted below in Figure 1c.

### 2.4. Image Analysis

Before image analysis with ImageJ, we performed a calibration using PhotoCap 6.0 software by adjusting luminance in the white zone to a common standard (RGB average value equal to 230). We used ImageJ to analyze the RGB color values of each zone before and after testing, and determined the mean intensity red values of each. We placed concentration values into a standard curve formula to determine estimated PQ and creatinine concentrations.

### 2.5. Clinical Samples

Human samples were collected at Chang Gung Memorial Hospital, Linkou, Taiwan. This study complied with the guidelines of the Declaration of Helsinki and was approved by the Medical Ethics Committee of Chang Gung Memorial Hospital.

## 3. Results and Discussion

### 3.1. PQ Standard Curve in Serum and Buffer Systems

**Figure 3:**
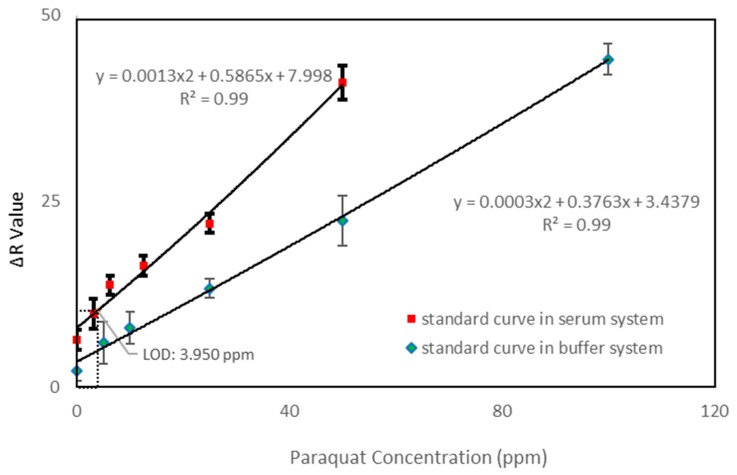
Paraquat standard curve (n = 8, N = 3). Green indicates the standard curve in a buffer system, with a concentration range of 0–100 ppm; red indicates the standard curve in a serum system, with a concentration range of 0–50 ppm. Correlation coefficient (R^2^) of standard curves in both buffer and serum systems equals 0.99.

Following our previous study [6], we elected to employ an ascorbic acid mechanism to produce Paraquat color differences. This mechanism is stable and reaction time was suitable to our device development. We deviated from the abovementioned study with regard to reagent application including an increase in the concentration of ascorbic acid from 5% to 10%. The standard curves shown in Figure 3 were created under these new conditions. The ΔR (delta red) value in Figure 3 is the red mean intensity difference in RGB analysis after 10 min minus the image background value. It represents the total change in red color. We gave image resolution considerable focus as an important aspect of developing an impactful colorimetric method test. This led us to follow an alternative approach to reagent placement that significantly influenced analyzed RGB values.

We provided a standard curve in both the buffer and serum systems. The concentration range of serum in our serum system was 0–50 ppm, and the concentration range of buffer in our buffer system was 0–100 ppm. Both systems demonstrated a correlation coefficient (R^2^) = 0.99, but the ΔR value between the systems indicated different tendencies. In our buffer system, the delta value was equal to the serum system level at a higher concentration. The slope of standard curve in our buffer system was gentle and the slope in our serum system was rather steep.

To determine the clinical viability of our device, we compared the serum system results of our paper-based tool with serum results obtained by standard clinical means. We found the standard curves from both to be comparable, and our device to be a suitable alternative. The detection limit (LOD) was determined at three standard deviations from zero along the standard curve.

### 3.2. Creatinine Standard Curve in Serum and Buffer Systems

Normal human serum creatinine levels range from 5.0–10.0 ppm for women, and from 7.0–12.0 ppm for men. Based on these values, we established a standard curve first in a buffer system, to ensure that the reaction on our 2-in-1 PADs was stable and that the device could be used for colorimetric methods. We then set up a standard curve in the serum system to assess feasibility and practicality of clinical usage. Based on the RGB intensity values from our creatinine reaction, we designed several experiments using different backgrounds, i.e., different materials behind our paper-based device, such as a bare desktop or white paper to determine which would provide the best colorimetric results.

**Figure 4:**
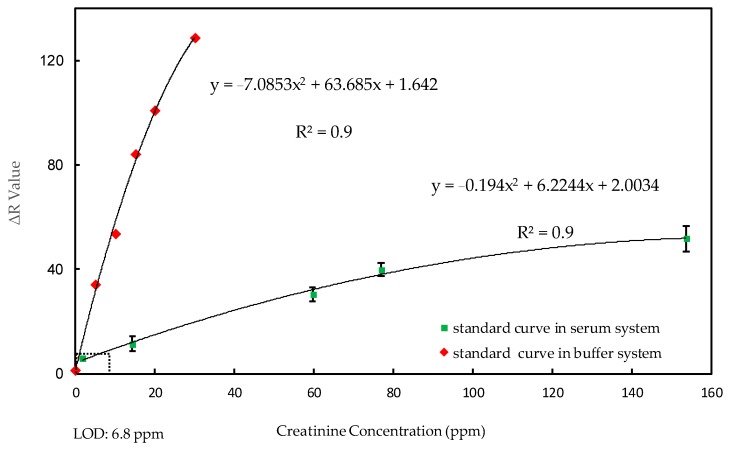
Creatinine standard curve (n = 8, N = 3). Red indicates the standard curve in a buffer system, with a concentration range of 0–30.0 ppm; green indicates the standard curve in a serum system, with a concentration range of 7.3–153.6 mg/dL. Correlation coefficient (R^2^) of standard curves in both buffer system and serum system equals 0.99.

Figure 4 demonstrates that creatinine concentration in our buffer system ranged from 0.0–30.0 ppm, and that serum ranged from 7.3–153.6 ppm. Because there is a basic, standard concentration of creatinine in serum, our standard curve for creatinine in serum does not start at 0 ppm. The SD value of our lowest concentration, 7.3 ppm, corresponded to 0.0549 mg/dL, and the LOD was determined at three standard deviations from the lowest concentration along the standard curve. Standard curves in both systems showed a correlation coefficient of 0.99, indicating a stable and accurate tendency.

**Figure 5:**
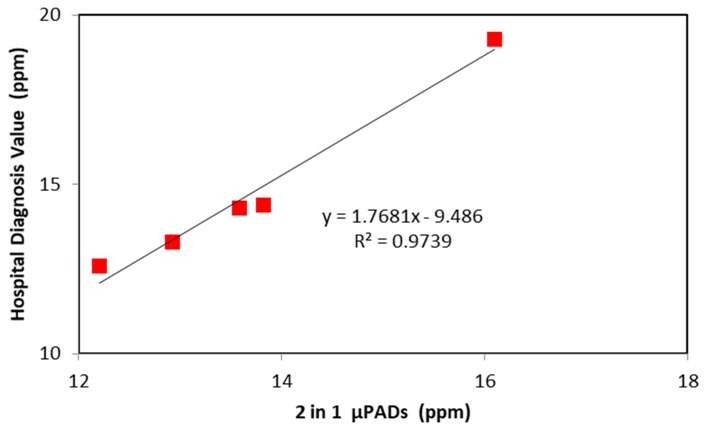
Linear regression analysis of creatinine detection, with the vertical axis indicating the clinical value of creatinine concentration, and the horizontal axis indicating the PAD-determined value of creatinine concentration.

Figure 5 shows a linear regression analysis of creatinine detection, indicating a high correlation between laboratory-determined values and values determined using our 2-in-1 PAD. The R^2^ of 0.9988 for our PAD is comparable to clinically obtained values.

### 3.3. 2-in-1 PADs Performance

Results from using our 2-in-1 PADs to detect serum paraquat and creatinine concentration for patient specimens (T1, T2, T3, T4, T5) provided by Linkou Chang Gung Memorial Hospital are shown in Table 1. Of the five patient specimens, each are clinically indicated to be suffering from paraquat poisoning without kidney failure. Based on the SIPP index, we determined that a PQ intoxication of 10 ppm was dangerous.

In the high concentration group (T1, T3, T4, T5), we saw that the color change in our PADs correlated with high concentration detection in all cases. For the single low concentration sample (T2), the difference between our PADs and that taken at the hospital was within 20%, indicating PADs diagnostic viability.

Due to normal creatinine values, we were unable to see obvious color differences using our PADs. The low creatinine concentration was detected by analyzed images and delta values found before and after the reaction. The differences between PAD and clinical values were under 20%. We did notice that patient T1 was suffering from a high poison intoxication level that induced serious hemolysis, which may have influenced PAD detection efficacy.

A total of five patients with PQ exposure were included in this study. Their clinical data are summarized in Table 2. All patients committed suicide by drinking PQ. Most patients were male elders. Four were considered severe intoxication patients because their serum PQ concentration was greater than 10 ppm. Apart from one patient who refused treatment, the other four patients were intensively treated with a standard detoxification protocol including charcoal hemoperfusion, pulse therapies with methylprednisolone and the cytotoxic agent (cyclophosphamide), and extended treatment with dexamethasone [3,11,35,36]. The calculated SIPP score was high (74.13 ± 94.16), and each patient had a SIPP greater than 10. Despite aggressive therapy, all patients (100%) died within 1–3 days of intoxication.

## 4. Conclusions

Based on our detection data, our 2-in-1 PADs performed comparably to the highest clinical standard with less complicated sample preparation. Paper-based analytical devices have advantages including low cost, disposability, carrying convenience, and they provide a platform to simultaneously detect and analyze multiple samples. In remote areas and areas with little or no clinical availability, PADs can be used to rapidly provide physiological indicators to on-site clinical staff, or transmitted to remote clinical staff to judge the status of the poisoned patient [37]. Potential future work based on this research could focus on the following three aspects: (i) accurate fluid control without pipetting errors; (ii) development of calibration standard curves for sera having different color characteristics; and (iii) a whole blood test that would be even more time- and cost-effective. This device can be considered a successful detection tool for clinicians with regard to saving time, cost, and patients lives.

## Figures and Tables

**Table 1:** Paraquat (PQ) and creatinine detection results from five PQ-poisoned patients comparing our detection methods and clinically determined values, which are considered to be the highest clinical standard.

2-in-1 μPADs		Paraquat (ppm)		Creatinine (ppm)	
		Our Value	Hospital	Our Value	Hospital
T1	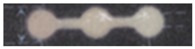	22.59	>10	16.10	19.3
T2	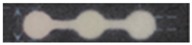	5.99	5.1	12.92	13.3
T3	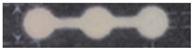	26.52	>10	13.82	14.4
T4	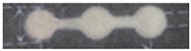	35.19	>10	13.58	14.3
T5	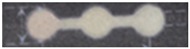	25.0	>10	12.20	12.6

**Table 2:** Clinical data of patients with PQ intoxication (n = 5).

Patient Number	Age	Sex	Time between Paraquat Ingestion and Hospital Arrival (h)	Blood Paraquat Level (ppm)	Severiry Index of Paraquat Poisoning (ppm)	Blood Creatinine Level (ppm)	Treatment	Duration of Hospitalization (day)	Outcome
T1	50	M	6	10	60	19.3	Hemoperfusion cytotoxic agent, glucocorticoid	1	Dead
T2	62	M	6	5.11	30.66	13.3	Hemoperfusion cytotoxic agent, glucocorticoid	1	Dead
T3	43	M	2	10	20	14.4	Hemoperfusion cytotoxic agent, glucocorticoid	1	Dead
T4	87	M	2	10	20	14.3	Hemoperfusion cytotoxic agent, glucocorticoid	1	Dead
T5	50	M	24	10	240	12.6	Hemoperfusion cytotoxic agent, glucocorticoid	3	Dead

Note: The severity index of paraquat poisoning was derived the product of plasma paraquat level in milligrams per liter and time from paraquat ingestion to arrival in hours.
